# Hints with Regard to the Natural History and Medicinal Properties of Veratrum Viride

**Published:** 1853-08

**Authors:** Isaac Branch

**Affiliations:** Abbeville, S. C.


Art. IV.—*Hints with regard to the natural history and medicinal properties of Veratrum Viride.* By Isaac Branch, M. D., of Abbeville, S. C.

This plant has recently become an important article of the materia medica, at least in the South, and it is for the purpose of introducing it into the North-west that we have consented to write this concise article. We have used this article extensively ourself, with the most satisfactory results, and believe that it is not only the privilege, but the imperious duty of every member of the profession to contribute his mite to the stock of general knowledge, so that the result of the experience of all may be placed in some tangible form before the professional world. Our remarks will be concise, and we trust to the point, and as they have been spread on several occasions before the profession in the South upon the same subject, we must be exceedingly brief on this occasion. Indeed, our peregrinations for the coming few months, will not permit us to write an extensive article. We will add that we have never sold an ounce of the article in our life, nor are we interested, in any manner whatever, in its sale.

At the instance and request of Dr. W. C. Norwood, of South Carolina, who is entitled to more credit for investigating this article than all of the rest of the profession combined, we have deposited, for him, a small quantity of the pure tincture in Nashville, Louisville, and Indianapolis. The formula for its preparation has been published to the world by Dr. Norwood, time and again, and for the informationof those who have not seen it, we will repeat it:

Root of Veratrum Viride, 1 lb.
Officinal Alcohol, 1 pint.

Macerate for about ten days, decant and strain, and it is fit for use. It may be thought that we use an unnecessary quantity of the root, but the object is to have a pure saturated tincture, and all we have to say in regard to its use and merits, contemplates a tincture of that kind, therefore, a little extra quantity of the root makes no difference, whilst a little short of the requisite quantity will materially affect the article. Any practitioner of medicine may prepare this article for use, provided he can procure a pure article of the root, and here rests the difficulty. If he orders the *Viride*, he is liable to procure the *Album*, and vice versa ; indeed, the endorsement upon the package procured, affords no security ; for the *Viride* is more apt to be labelled *Album* than otherwise. We will explain this confusion.

The *Veratrum Viride* is an American plant, whilst the *Veratrum Album* is exclusively and entirely European. Until comparatively speaking quite recently, they were regarded even by botanists as identical, and the same identical medicinal qualities ascribed to each. In external appearance they somewhat resemble each other, whilst in medicinal properties they differ widely.

We regard this article as about as important in the treatment of typhoid and other affiliated diseases, as the sulphate of quinine is in the treatment of intermittent and remittent fevers. We are aware that this is assigning to the article under consideration a very prominent position—it is placing it upon the top shelf, but our experience justifies the remark. Although we have never used this remedy in a pure case of typhus, indeed we have never seen such a case at the South, we believe, reasoning from analogy, that it is as well adapted to the treatment of typhus as it is to typhoid diseases. In any case of disease with a hot and dry skin, and a frequent pulse, we should most assuredly resort to its use, guarded by the views we shall advance in the sequel The article is peculiarly adapted to the treatment of asthenic diseases—it diminishes the *frequency* of the pulse, whilst it does not reduce the *strength*; indeed, we have very often seen the strength considerably increased under its use.

Our plan for using the article in typhoid diseases is as follows, and we commence our treatment at our first interview with the patient: we never resort to any preparatory treatment. In a case of typhoid fever or typhoid pneumonia, presenting the ordinary symptoms, if the patient be an able-bodied, athletic adult, we commence the treatment of the case with from five to seven drops of the tincture, giving it at intervals of two hours, increasing a drop at each succeeding dose, until the pulse is reduced to the desired standard as to frequency, which will generally be the case in from eight to twelve hours, and here we hold the pulse with more or less of the article, giving it at regular intervals until the disease is broken up. We have often taken a case with a hot, dry and husky skin, a dry tongue, great restlessness, with a pulse of 140 in a minute, and within the course of twelve hours we have had a cool skin, a moist surface, a tranquil condition of the system, with a pulse of from 60 to 80 beats in a minute. The only bad effect which ever results from the use of the article, is excessive nausea, retching, and vomiting, and there is no difficulty in guarding against this, if small doses are used in the beginning of the treatment, and increased as above detailed. After the pulse is brought under the control of the article, three or four drops will frequently hold it there if repeated every two or three hours. There are cases of typhoid diseases which require anodynes from the commencement of the attack, in which cases we usually give a tea-spoonful, more or less, of paregoric, or an equivalent quantity of opium or morphine, in some other form, with each dose. We usually give a blue pill, or some other eccoprotic—we never purge.

If the patient be a feeble lady, or a child, we give less of the article, according to circumstances, until the susceptibility of the patient is ascertained. We have never used the article in scarlet fever, but can have no doubt of its beneficial effects in such cases. We ask the profession in the Northwest to try it. We have not time to continue our remarks.

